# Functional metabolite reserves and lipid homeostasis revealed by the MA-10 Leydig cell metabolome

**DOI:** 10.1093/pnasnexus/pgac215

**Published:** 2022-09-27

**Authors:** Prasanthi P Koganti, Lan N Tu, Vimal Selvaraj

**Affiliations:** Department of Animal Science, College of Agriculture and Life Sciences, Cornell University, Ithaca, NY 14853, USA; Department of Animal Science, College of Agriculture and Life Sciences, Cornell University, Ithaca, NY 14853, USA; Department of Animal Science, College of Agriculture and Life Sciences, Cornell University, Ithaca, NY 14853, USA

**Keywords:** cholesterol, lipid, steroidogenesis, metabolism, Leydig cells

## Abstract

In Leydig cells, intrinsic factors that determine cellular steroidogenic efficiency is of functional interest to decipher and monitor pathophysiology in many contexts. Nevertheless, beyond basic regulation of cholesterol storage and mobilization, systems biology interpretation of the metabolite networks in steroidogenic function is deficient. To reconstruct and describe the different molecular systems regulating steroidogenesis, we profiled the metabolites in resting MA-10 Leydig cells. Our results identified 283-annotated components (82 neutral lipids, 154 membrane lipids, and 47 other metabolites). Neutral lipids were represented by an abundance of triacyglycerols (97.1%), and low levels of cholesterol esters (2.0%). Membrane lipids were represented by an abundance of glycerophospholipids (77.8%), followed by sphingolipids (22.2%). Acylcarnitines, nucleosides, amino acids and their derivatives were the other metabolite classes identified. Among nonlipid metabolites, we recognized substantial reserves of aspartic acid, choline, creatine, betaine, glutamine, homoserine, isoleucine, and pantothenic acid none of which have been previously considered as a requirement in steroidogenic function. Individually limiting use of betaine, choline, or pantothenic acid, during luteinizing hormone-induced steroidogenesis in MA-10 cells resulted in substantial decreases to acute steroidogenic capacity, explained by intermediary metabolite imbalances affecting homeostasis. As such, our dataset represents the current level of baseline characterization and unravels the functional resting state of steroidogenic MA-10 Leydig cells. In identifying metabolite stockpiles and causal mechanisms, these results serve to further comprehend the cellular setup and regulation of steroid biosynthesis.

Significance Statement Leydig cells are known for their tremendous demand for cholesterol during acute de novo biosynthesis of the male hormone. Looking beyond the essential cholesterol storage and mobilization, we uncover the molecular composition of MA-10 Leydig cells and describe different metabolic systems that support steroidogenic function. Functional testing via limiting use of specific metabolite stockpiles identified crucial components and novel paths to support steroid biosynthesis. Our findings build a metabolomic framework for steroidogenesis and provide new fundamental insights into its biosynthetic regulation.

## Introduction

Steroidogenic cells are specialized endocrine cells capable of synthesizing steroid hormones via a multi-step enzymatic bioconversion of cholesterol (1, 2). At the cellular level, the rate of steroidogenesis is precisely regulated by the essential step of mitochondrial cholesterol import, which is the passage of cholesterol from the outer to inner mitochondrial membrane (3, 4). Biosynthesis of the first steroid pregnenolone occurs within the mitochondria via the action of CYP11A1 present at the matrix side of the inner mitochondrial membrane (5–7). Periodic trophic stimulation that demands acute responses in steroidogenic cells is a predominant aspect of systemic steroidogenic homeostasis in vivo. In testicular Leydig cells, pulsatile pituitary release of luteinizing hormone (LH) induces periodic testosterone elevation (8). Consequently, steroidogenic cells have high intermittent functional demand for cholesterol substrate (9–12). In addition to free cholesterol (FC) that is readily available (13, 14) and extracellular sources in the form of plasma/interstitial lipoproteins (15, 16), lipid droplets (LDs) as a cellular cholesterol store are a core characteristic consistent across steroidogenic cells of the adrenals, ovaries, and testes (17–20). Utilization of cholesterol esters (CEs) stored in LDs and resulting depletion are known to occur after trophic stimulation of steroid hormone biosynthesis (12, 21). In the absence of extracellular sources, depletion of CEs stored in LDs has been demonstrated to make Leydig and adrenocortical cells refractory to subsequent trophic stimulation (11, 22, 23), underscoring the functional significance of cholesterol storage and the importance of CE/LDs in steroidogenic cells (12, 24–26). Additionally, an extent of de novo cholesterol synthesis is known to be triggered to ensure full steroidogenic capacity in Leydig cells (13, 27).

Studies on steroidogenic cells have rarely extended beyond direct emphasis on cholesterol metabolism and storage. From the early 1970s, studies have recorded changes in membrane lipids and altered CE metabolism during the Leydig cell response to gonadotropins (21, 28). More recently, mass spectrometry-based approaches have been used to profile dynamic changes to lipid composition in the different organelle compartments (29), or examine lipidome changes in response to specific pharmacological agents (30). However, there are presently no studies that profile nonlipid metabolites for systems biology analyses in any steroidogenic cell type. Previous investigations on nonlipid metabolites have been targeted to examine limited substrates for cholesterol synthesis (31, 32) or energy metabolism (33, 34), and have not extended to uncover any networked metabolic pathways or interactions.

In this study, we examine the metabolome of steroidogenic MA-10 Leydig cells and quantitatively profile both lipid and nonlipid metabolites with an objective of uncovering both the identity and possible roles of different stored metabolites that might have supportive functions in steroidogenesis. By using reverse-phase liquid chromatography coupled with electrospray ionization (ESI) quadrupole time-of-flight mass spectrometry, we annotated metabolites to set a baseline quantitative profile. These data uncovered previously unknown functional components and metabolite reserves relevant for the acute regulation of steroidogenesis.

## Results

### MA-10 Leydig cell metabolite landscape

**Fig. 1.**
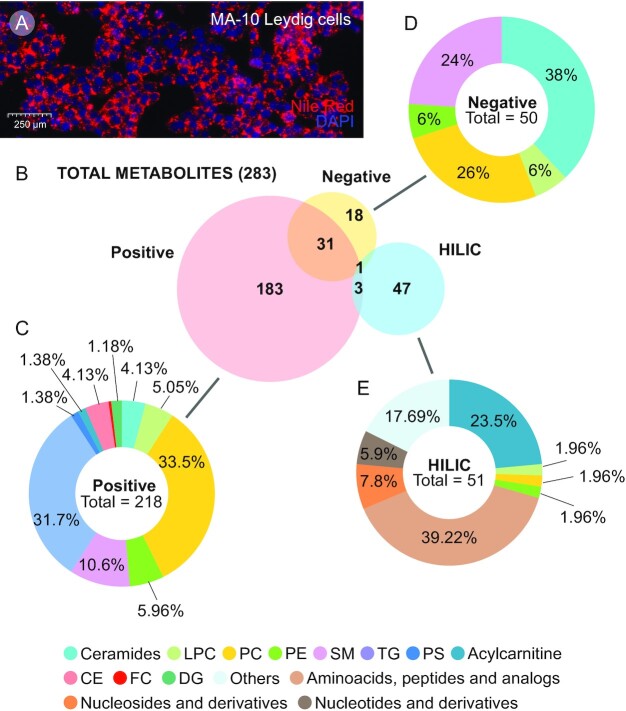
Leydig cells and the classification of its metabolites. (A) Lipid droplets in steroidogenic cells are considered characteristic of their function. Representative image shows MA-10 Leydig cells in culture with lipid droplets visualized by fluorescence of Nile Red staining (Red). Nuclei are counter-stained with DAPI (Blue). (B) Venn diagram showing the distribution and overlap of the total metabolites identified in positive [ESI(+)], negative [ESI(−)] and HILIC modes in the metabolomics approach. (C to E) Donut charts showing the relative abundance (as percentages) of the different metabolite groups/categories identified within each detection mode in the metabolomics approach.

Lipid storage and associated cholesterol mobilization is of physiological significance in Leydig cell function. Recapitulating baseline homeostasis in the absence of trophic stimulation, MA-10 Leydig cells contain LDs as observed using Nile Red staining of resting cells (Fig. 1A). Establishment of the baseline/resting metabolic profile of these cells identified a total of 283 unique metabolites annotated using LipidBlast; identification and annotation was performed in electrospray ionization (ESI) (+/−) and hydrophilic interaction chromatography (HILIC) ESI (+) assays (Fig. 1B). Of this total, 218 metabolites were identified in ESI (+), 50 in ESI (−), and 51 in HILIC ESI (+) assays. Identification was numerically highest in the ESI (+) assay and accounted for 77% of the total metabolites annotated, with 65% of them exclusively identified in this assay. Metabolites identified exclusively in ESI (−) and HILIC ESI (+) assays accounted for 6.4% and 16.6%, respectively (Fig. 1B). Identification of some metabolites were redundant as they occurred in more than one assay; 11% were identified in both ESI (+) and ESI (−) assays, 1.1% in ESI (+) and HILIC ESI (+), and 0.35% in all three assays. Redundancies were examined for relative quantitative consistency, and the mode with the least standard deviation was retained.

Metabolites in each assay were further classified; the percentages of each metabolite species identified in specific assays are shown in relation of the full dataset for the different assays (Fig. 1C to E). Phosphatidylcholine (PC) and triacylglycerol (TG) were the major lipid classes identified in ESI (+), and together accounted for 65% of the annotated metabolites in this assay; in addition, a variety of CEs were also annotated in the ESI (+) mode. Different species of membrane lipids were represented in the ESI (−) assay: ceramide (CM), sphingomyelin (SM), and phosphatidyl choline (PC) that accounted for 88% of the identified metabolites in this mode. Approximately one-fourth of the metabolites in HILIC ESI (+) assay were acylcarnitine (AC), which accounted for 23.53% of the total identified metabolites. Besides lipids, other metabolites that were identified in HILIC ESI (+) assay were amino acids, peptides, and their analogs (39.2%), nucleoside (7.8%), and nucleotide derivatives (5.9%). The full list of metabolites identified is provided in Supplementary Material File 1.

### CE diversity and neutral lipids

**Fig. 2.**
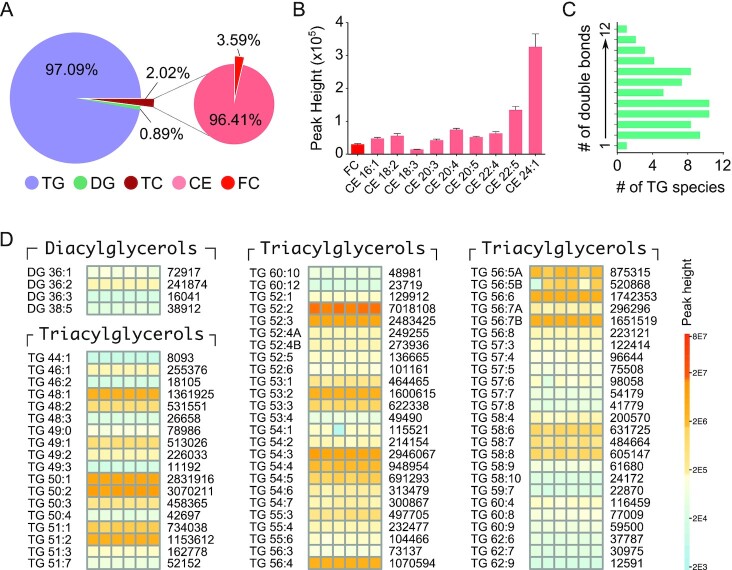
Neutral lipid diversity in Leydig cells. (A) Categories of neutral lipids identified in Leydig cells as pie charts. Triacylglycerols (TG) are the most predominant neutral lipid species (97.09%). Total cholesterol (TC) formed 2.02% of neutral lipids in Leydig cells, with 96.41% stored as cholesterol esters (CE). As minor proportions, diacylglycerols (DG), and free cholesterol (FC) are shown. (B) Expression levels of FC and different CE species in Leydig cells. CE24:1 was found highly abundant above all other CE species. (C) Degree of unsaturation observed in all identified TG species. (D) Quantitative heatmap showing diversity and expression levels of all DG and TG species identified in Leydig cells. Average peak heights are indicated on the right.

In the breakdown of neutral lipids [TG, diacylglycerol (DG), and total cholesterol (TC)], MA-10 Leydig cells contained 97.1% of TG, 0.9% DG, and 2.0% TC (Fig. 2A). In TC, levels of free cholesterol (FC) were markedly lower (3.6%), when proportionately compared to CE levels (96.4%). When represented as a ratio, CE:FC was 26.9:1; this proportional difference is quantitatively substantial. With CEs, nine species were identified (Fig. 2B); the most abundant CE species was CE 24:1 followed by CE 22:5, both constituting 56.8% of the total CE content. The number of polyunsaturated CE species (*n* = 7) was more than the monounsaturated CE species (*n* = 2). The ratio of TG:CE was 50:1, markedly lower compared to TG:FC 1337.5:1. Of the TGs, most of the species identified were polyunsaturated, containing up to 12 double bonds (Fig. 2C), a trend that was also seen in DGs. The segment of DGs was substantially lower than TG and TC, represented by only four species. The absolute levels of DG and TG species are presented as heatmaps (Fig. 2D). The abundance of DG species was in the order of DG 36:2 > DG 36:1 > DG 38:5 > DG 36:3. The storage form as TG was the most abundant form of neutral lipid present in MA-10 Leydig cells. Sixty-eight TG species were identified in MA-10 Leydig cells, and TG 52:2 > TG 50:2 > TG 54:3 > TG 50:1 > TG 52:3 > TG 56:6 > TG 56:7B > TG 48:1 > TG 51:2 > TG 56:4, could be considered the most abundant. Only one of the 68 TGs identified was saturated (TG 49:0), with 9 being monounsaturated and 58 polyunsaturated.

### FC and membrane lipids

**Fig. 3.**
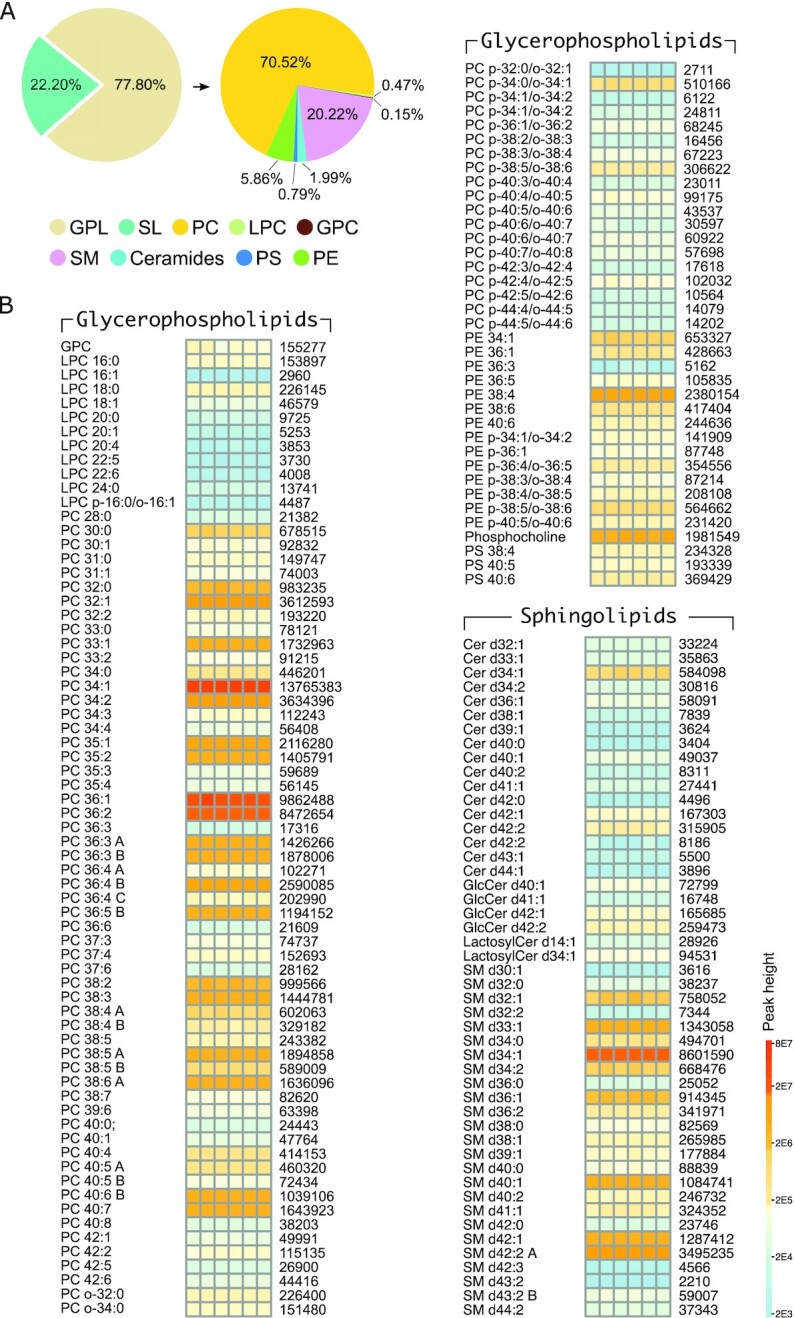
Membrane lipid diversity in Leydig cells. (A) Categories of membrane lipids identified in Leydig cells as pie charts. Glycerophospholipids (GPL) represent 77.8% of membrane lipids identified, with the remainder being sphingolipids (SL). Further classification of the different GPL and SL are also shown on the basis of relative percentages for phosphatidylcholine (PC), lysophosphatidylcholine (LPC), glycerophosphatidylcholine (GPC), sphingomyelin (SM), ceramides, phosphatidylserine (PS), and phosphatidylethanolamine (PE). (B) Quantitative heatmap showing diversity and expression levels of all GPL and SL species identified in Leydig cells. Average peak heights are indicated on the right.

A total of 154 membrane lipids were identified and further classified as glycerophospholipids (GPLs) and sphingolipids (SLs) in MA-10 Leydig cells. The number and concentration of GPLs [phosphotidylcholine (PC), phosphatidylethanolamine (PE), phosphatidylglycerol (PG), lysophosphatidylcholine (LPC), and phosphatidylserine (PS)] were higher than SLs (SM, CMs, and glycospingolipids–glucosylceramide and lactosylceramide), in a 3.5:1 ratio (77.8% and 22.2%, respectively) (Fig. 3A). The list consisted of 106 different GPLs and 49 different SLs identified. Membrane lipid PC (70.5%) was the most dominant GPL with 77 different PC species identified. The second most abundant membrane lipid was SM (20.2%) followed by PE (5.86%), CMs (1.99%), PS (0.79%), LPC (0.47%), and GPC (0.15%) (Fig. 3A).

The different GPLs and their absolute levels are presented as a heatmap (Fig. 3B). Among PCs, PC 34:1 showed very high abundance, followed by PC 36:1 > PC 36:2 > PC 34:2 > PC 32:1 > PC 36:4B > PC 38:4 > PC 35:1 > Phosphocholine > PC 38:5A > PC 36:3B > PC 33:1 > PC 40:7 > PC 38:6A > PC 38:3 > PC 36:3A > PC 35:2 > PC 36:5B > PC 40:6B, which were all relatively abundant (Fig. 3B). Among PEs, PE 38:4 was the only PE with high expression (Fig. 3B). The different SLs and their absolute levels are also presented as a heatmap (Fig. 3B). The number and levels of SM were higher than CMs. Among SMs, SM d34:1 > SM d42:2A > SM d33:1 > SM d42:1 > SM d40:1, were relatively high in the order of abundance detected. Among CMs, cer d34:1 > cer d42:2 > cer d42:1, were relatively high the order of abundance detected. When considered as a subset of total metabolites detected, the percentages of GPL and SL were 55% and 15.7% respectively. The ratios of GPL:FC and SL:FC were 2595:1 and 740:1, respectively, with both being significantly higher than the ratio of TG:CE (50:1).

### Other functional metabolites

**Fig. 4.**
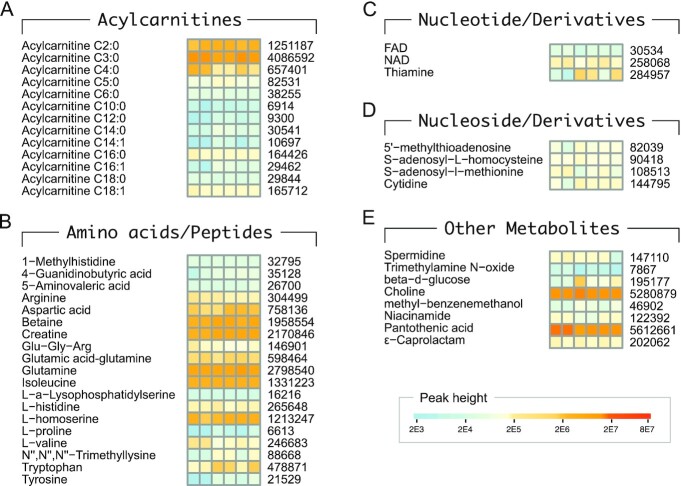
Acylcarnitines and other metabolites identified. Quantitative heatmaps showing absolute levels of: (A) acylcarnitines, (B) amino acids, peptides and their derivatives, (C) nucleotide and their derivatives, (D) nucleoside and their derivatives, and (E) other metabolites, as identified in Leydig cells. Average peak heights are indicated on the right.

ACs, amino acids/peptides, nucleotide, and nucleoside derivatives, and other uncategorized metabolites were other functional metabolites identified in MA-10 Leydig cells (Fig. 4A to E). Thirteen ACs, that included short, medium, and long chains ranging from C2 to C18 were identified (Fig. 4A). Most ACs were saturated (*n* = 10) with three being monounsaturated (*n* = 3). All the short-chain ACs identified were saturated, with three of them: AC 3:0 > AC2:0 > AC4:0, being the most predominant. For amino acids and peptides, glutamine > creatine > betaine > isoleucine > L-homoserine, were of very high abundance, and aspartic acid > glutamic acid-glutamine > tryptophan > arginine > L-histidine > L-valine were also abundant (Fig. 4B). For nucleotide and derivatives, thiamine > NAD, were abundant (Fig.   4C). For nucleoside and derivatives, cytidine > S-adenosyl-l-methionine, were abundant (Fig. 4D). Among other metabolites, pantothenic acid > choline, were of very high abundance, and caprolactam > ß-d-glucose > niacinamide were also abundant (Fig. 4E). The metabolites in this category and the majority of AC were identified only in HILIC assay.

### Inhibiting utilization of pantothenic acid blocked steroidogenic capacity

**Fig. 5.**
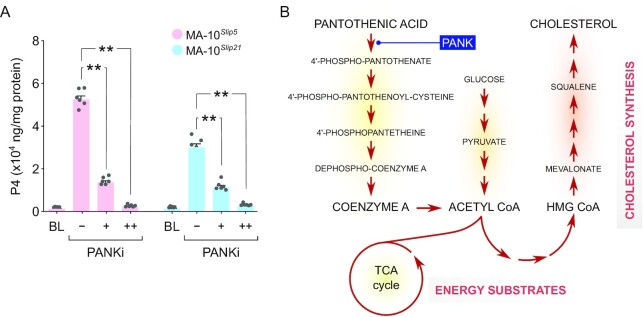
Inhibition of pantothenic acid utilization significantly decreased the steroidogenic capacity of Leydig cells. (A) In evaluating the rate of steroid hormone biosynthesis (Progesterone/P4 production) after trophic stimulation (using hCG; BL - baseline) in MA-10 cell clones, a dose-dependent decrease in P4 was observed in concurrent treatment with a pantothenate kinase inhibitor (PANKi) (n=6; *P* < 0.01 at both 10 µM [+] and 100 µM [++] inhibitor concentrations). (B) Key bioconversions associated with pantothenic acid metabolism and pathways that might impact processes such as synthesis of cholesterol and energy substrates relevant to supporting steroidogenesis. The step of PANK inhibition is indicated.

Pantothenic acid was one of the metabolites of extremely high abundance detected under the uncategorized metabolites (Fig. 4E). Treating LH-responsive MA-10 Leydig cell clones with a pantothenate kinase inhibitor (PANKi) during induction of steroidogenesis with hCG almost completely blocked steroid hormone biosynthesis in a dose-dependent manner. The amount of progesterone produced was drastically decreased with PANKi (10μM and 100μM), by 73.5% and 95.6% in MA-10*^Slip5^* cells, and 64.3% and 90.56% in MA-10*^Slip21^* cells, respectively (Fig. 5A). Pantothenic acid is a precursor for CoA synthesis and formation of acetyl CoA influences both energy metabolism and cholesterol synthesis (Fig. 5B).

### Inhibiting utilization of betaine or choline limited steroidogenic capacity

**Fig. 6.**
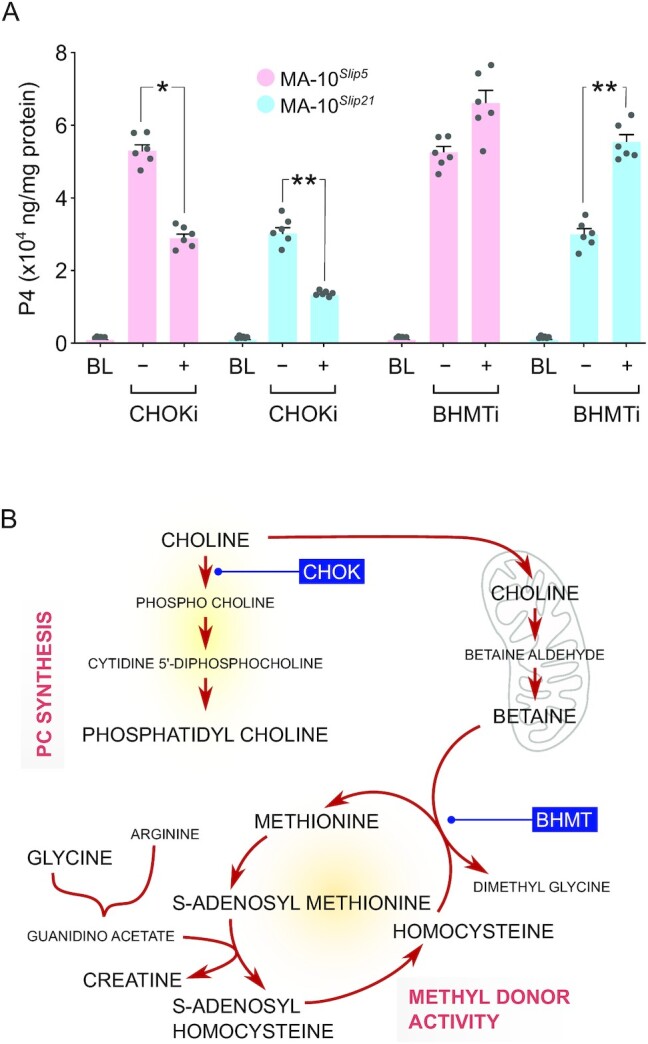
Inhibition of choline and betaine utilization significantly shifted the steroidogenic capacity of Leydig cells. (A) In evaluating the rate of steroid hormone biosynthesis (Progesterone/P4 production) after trophic stimulation (using hCG; BL - baseline) in MA-10 cell clones, decrease in P4 was observed in treatment with a choline kinase inhibitor (CHOKi; V-11-0711, 100 µM) (n=6; *P* < 0.01); increase in P4 was observed in treatment with a betaine-homocysteine S-methyltransferase inhibitor (BHMTi; CBHcy, 100 µM), but this was only significant in MA-10*^Slip21^* cells (n=6; *P* < 0.01), but not in MA-10*^Slip5^* cells. (B) Key bioconversions associated with choline metabolism and pathways that might impact processes such as phosphatidylcholine/PC synthesis, and methyl donor activity relevant to supporting steroidogenesis. The steps of CHOK and BHMT inhibition are indicated.

Betaine and choline were two of the metabolites of high abundance detected under amino acids (Fig. 4B) and uncategorized metabolites (Fig. 4E), respectively. Treating LH-responsive MA-10 Leydig cell clones with a choline kinase inhibitor (CHOKi) during induction of steroidogenesis with hCG suppressed steroid hormone biosynthesis (Fig. 6A). The amount of progesterone produced significantly decreased with CHOKi treatment by 45.8% and 54.7% in MA-10*^Slip5^* and MA-10*^Slip21^* cells, respectively. In contrast, treatment with BHMTi during induction of steroidogenesis with hCG increased progesterone levels by 23% and 83.5% in MA-10*^Slip5^* and MA-10*^Slip21^* cells, respectively (Fig. 6A). However, the increase was found to be significant only in MA-10*^Slip21^* but not in MA-10*^Slip5^* cells. Choline is a precursor for PC synthesis and also for the formation of betaine in the mitochondria; betaine acts as a methyl donor in the methionine-homocysteine cycle (Fig. 6B).

### Regulatory architecture of metabolic pathways is conserved in MA-10 cells

Comparing the transcriptome of MA-10 and primary Leydig cells revealed the roughly conserved pattern of gene expression specific to the metabolic pathways studied. Gene expression heat maps associated with cholesterol, FA, SL, and GPL metabolism are shown in Fig. S1. Gene expression heat maps associated with glycolysis, TCA cycle, choline, and SAM, CoA, amino acids, and other metabolites are shown in Fig. S2. From this mapping, it was visualized that pathways being dissected were comparably represented in both MA-10 and primary Leydig cells. There were two exceptions that we noted. First is the expression of glutamine synthase (*Glul*), that catalyzes the synthesis of glutamine from glutamate and ammonia; MA-10 cells seem to downregulate Glul expression (0.34 ± 0.06 CPM), compared to primary Leydig cells (4438.7 ± 281.8 CPM). As the nonessential amino acid glutamine is supplemented in the MA-10 culture medium, we suspect that this downregulation of glutamine synthesis might be a negative feedback or epigenetic anomaly that occurred in these cells. Second is the expression of ethanolamine kinase 2 (*Etnk2*), that catalyzes the first step in PE biosynthesis via the cytidine diphosphate (CDP) ethanolamine pathway; MA-10 cells seem to downregulate *Etnk2* expression (0.29 ± 0.04 CPM), compared to primary Leydig cells (90.5 ± 9.1 CPM). However, as *Etnk1* and *Etnk2* are both capable of phosphorylating ethanolamine (35), and *Etnk1* expression appears compensatory in MA-10 cells (143.8 ± 4.4 CPM) compared to primary Leydig cells (111.6 ± 8.6 CPM), the impact of this difference might be negligible. All other genes in the glutamine pathway and PE pathway were comparable, indicating that these two differences may not have a major influence on functional metabolites.

## Discussion

Analysis of low-molecular weight metabolites in a cell can inform on a variety of cellular components and biological processes (36, 37). Of the different metabolites, cholesterol and lipids are of particular significance in steroidogenic cells as they serve to sustain homeostasis and regulate substrate availability for steroid hormone biosynthesis. Numerous studies have evaluated cholesterol storage and mobilization together with the influence of TGs in this homeostasis (28, 38–41). A recent study has also evaluated the dynamics of lipid compositional changes in different cellular compartments relative to protein content in steroidogenic MA-10 cells (29). However, the integrated network on nonlipid metabolites and their role in steroidogenic functional homeostasis have remained largely unstudied.

In this manuscript, we investigate the baseline metabolome of MA-10 Leydig cells with an objective of evaluating the state of metabolic readiness for an acute functional event. This approach, which mimics the baseline Leydig cell state in vivo between episodes of LH pulses, unraveled the preparedness in the form of metabolite stockpiles that can promote steroid biosynthesis. Our results that present a coordinated network of lipid and nonlipid metabolites represents core metabolic paths for functional facilitation of the steroidogenic process.

### Cholesterol homeostasis

Most early work on cholesterol stored as CEs has been performed in adrenocortical (42), and ovarian granulosa and luteal cells (41, 43). In composition, studies have indicated multi-fold higher levels of CE compared to FC in these steroidogenic cells. In contrast, estimations in Leydig cells have been inconsistent; initial studies indicated that they contain 3 to 4-fold more FC than CE (44, 45). These results have since been amended in subsequent reports suggesting that in fact CE levels are ∼7-fold higher than FC (46), or that FC and CE levels are approximately equal (47). In our quantitation, we find that CE levels are 27-fold higher than FC in resting Leydig cells, mirroring storage proportions reported in adrenocortical and ovarian steroidogenic cells. Moreover, the preferentially accumulated and mobilized CE species reported for ovarian steroidogenic cells [CE 20:4, 22:4, 22:5] (48, 49), and adrenocortical cells [CE 20:4, 22:4] (50), were also represented in the MA-10 Leydig cell metabolome. The polyunsaturated nature of these CE appears characteristic of steroidogenic cells. This propensity is perhaps indicative of a specific mechanism/regulation for packing and/or mobilization of high levels of CE in steroidogenic lipid droplets.

### Triglycerides and fatty acids

The CE hydrolysis mediated by hormone sensitive lipase (HSL) to increase FC levels in MA-10 cells (51) also results in the release of free fatty acids (FFAs). The HSL can also work to release FFAs from stored TG (52–55). However, the disposition of FFAs in steroidogenic cells is not completely understood. For a functional response in energy metabolism, FFA released enters the fatty acid oxidation pathway. Intriguingly, an inverse relationship was observed between the concentration of nonesterified long-chain unsaturated fatty acids, TGs, and testosterone production (56, 57). However, not all nonesterified long chain unsaturated fatty acids have similar effect on steroidogenesis; the fatty acids: oleic, stearic and linoleic acids could inhibit steroidogenesis, whereas palmitic acid could stimulate hormone production (58, 59). Besides, such reduction in hormone production was not observed when cells were provided with 22(R)-hydroxycholesterol (58), indicating that these fatty acids might have roles linked to cholesterol trafficking (60). Our results show that 85.3% of TG are polyunsaturated, indicating a preferential storage, and upon mobilization, has the potential for targeted regulation of cholesterol trafficking as FFA. Such secondary regulation could also be coupled to CE acquisition and processing differences observed between different steroidogenic tissues such as the adrenocortical cells, ovaries, and testicular Leydig cells (61, 62). Similarly, studies on comparative physiology have also indicated disparate mechanisms between steroidogenic systems that could also be impacted by TGs and FFAs in regulating cholesterol storage and use (63–65). The in vivo relevance of such multimodal regulation of Leydig cell steroidogenesis by different combinatorial lipid species remains to be investigated.

### Membrane lipids

The percentage of membrane components GPL and SL accounted for 70% of the total lipids in Leydig cells. Rapid increases to GPL, i.e. PC, PE, PG, and PI have been reported in response to trophic stimulation of Leydig cells (66), adrenocortical cells (67–69), and ovarian luteal cells (70). Therefore, the baseline abundance of GPL might be an adaptation in steroidogenic cells that would enable the handling of a surge in FC after stimulation and facilitate transport to the mitochondria for steroidogenesis. Moreover, certain species of unsaturated GPLs are known to stimulate steroid hormone production; an activity that is lost by hydrogenating the unsaturated bonds (71). We document a much elaborate profile for unsaturated PC and PE in Leydig cells, but their poised activities remain to be fully elucidated.

Roles for SL in directly supporting (72, 73) or antagonizing (74) specific enzymatic steps in steroidogenesis have been demonstrated in adrenocortical cells. But as a direct impact, it is well known that cholesterol SM association in the plasma membrane (75) is important for homeostasis. Forced breakdown of SM using sphingomyelinase has been demonstrated to decrease the cholesterol-holding capacity of the membranes and increase steroid hormone production (76). We have previously noted a similar cholesterol-SM association in metabolomics performed in mice fed a high cholesterol diet (37). Similarly, CMs have been reported to enhance steroidogenesis in Leydig cells (77, 78), but an antagonizing function for CMs via suppression of steroidogenic acute regulatory protein (STAR) expression has also been reported in Leydig cells (79). Extensive compositional remodeling of membrane compartments during acute steroidogenesis in MA-10 Leydig cells (29), perhaps sets up the path to cholesterol trafficking for facilitating function.

### Acylcarnitines

Elevated physiological levels of AC C3 and AC C2 in Leydig cells might be indicative of a mechanism that regulates glucose homeostasis and restricts metabolic flexibility (80). The enzyme carnitine O-acetyltransferase (CRAT) that resides in the mitochondrial matrix is known to selectively act on short-chain acyl-CoAs (81–84). In parallel, although speculative, it is possible that AC C3 and AC C2 elevation is indicative of a mechanism to combat a possible fatty acid stress, allowing for mitochondrial exit of acetyl motifs, preventing the inhibition of pyruvate dehydrogenase and incomplete fatty acid oxidation (85). This consideration is highly relevant for steroidogenic cells as substantial TG stored in steroidogenic cell lipid droplets require a rapid disposition as acute FFA release that is known to happen with trophic stimulation (86, 87).

### Amino acids

In the realm of amino acids, our results identify abundant levels of aspartic acid, glutamine, and isoleucine in Leydig cells. For aspartic acid, its accumulation, and a relationship to increasing testosterone production, has been previously observed (88). Although the mechanism remains unclear, increases in STAR protein levels have been noted with aspartic acid supplementation during steroidogenic stimulation in Leydig cells (89). For glutamine, its effectiveness as a metabolic fuel to sustain steroidogenesis with efficiencies comparable to d-glucose has been previously observed (90). Moreover, a glutamine–glutamate interchange could be characteristically driven by ferredoxin (Fdx1; also known as adrenodoxin) that results in transfer of electrons from Nicotinamide adenine dinucleotide phosphate (NADPH) to Fdx1 (91), which in turn reduces members of the mitochondrial cytochrome P450 family of proteins such as P450scc (CYP11A1) (92). There is no prior report linking isoleucine to steroidogenic efficiency. However, based on its abundance, it likely offers paths to increased levels of acetyl CoA for de novo cholesterol synthesis and energy metabolism (93, 94), and facilitate acute steroidogenesis.

### Metabolic regulation by pantothenic acid

High abundance of pantothenic acid was functionally corroborated in our results that inhibition of PANK could almost completely block progesterone production. We believe that this could be due to two reasons (Fig. 5B): pantothenic acid is a key precursor for the biosynthesis of coenzyme A/CoA (95, 96), an obligate cofactor of numerous enzymes that affect cellular intermediary metabolism, that includes synthesis of fatty acyl-CoA thioesters, the first step in intracellular metabolism of fatty acids (97). Moreover, CoA is crucial for the synthesis of acetyl CoA that is essential for de novo cholesterol synthesis in steroidogenic cells (98), and the tricarboxylic acid cycle noted to be important for providing energy substrates for steroid biosynthesis (93). An early study that examined the distribution of pantothenic acid in vivo using a rat model indicated adrenals as a site of high accumulation (99). Supplementing pantothenic acid in rats was shown to significantly increase the steroidogenic capacity of the adrenal cortex when stimulated with Adrenocorticotrophic hormone (ACTH) (100, 101). Our findings indicate that resting Leydig cells hold reserve pantothenic acid to support a steroidogenic function upon stimulation.

### Metabolic regulation by choline and betaine

Another metabolite, choline, identified in high abundance has not been previously observed or functionally examined in steroidogenic cells. The role of choline in synthesis of PC is well established (102). CHOK is known to be activated by cAMP-PKA signaling (103), suggesting that trophic stimulation via LH can increase PC synthesis (104). This is aligned with high levels of phosphocholine, and downstream components in the synthesis of PC, also detected as highly abundant in Leydig cells. Inhibition of choline kinase activity could substantially decrease steroidogenesis, indicating a crucial functional need for this choline reserve, via a mechanism that is likely explained by requirements for PC synthesis that would also support cholesterol transport (105).

Distinct from the choline contribution to PC synthesis, mitochondrial entry, and oxidation of choline to betaine, was also seen in high abundance, indicative of an active methyl donor program (106, 107). Methyl group in betaine is used by BHMT to convert homocysteine to methionine. Inhibiting BHMT activity could result in substantial homocysteine accumulation; reserves for S-adenosylhomocysteine (SAH) and homoserine, direct yet independent precursors of homocysteine synthesis were found to be abundant. It has been demonstrated that high levels of homocysteine can induce cholesterol biosynthesis (108), via activating cAMP-PKA signaling and transcriptional activation via sterol regulatory element-binding protein-2 (SREBP2) and cAMP response element-binding protein (CREB) (109). Action through this mechanism could be synergistic with trophic activation, and explain the significant increase in Leydig cell steroidogenesis observed with BHMT inhibition. Linked to this regulation, abundant SAH correlated with high levels of creatine detected (110), perhaps indicating preparedness for acute steroidogenesis that can be an energy-intensive process. High levels of creatine kinase and functional involvement of creatine in maintaining adenosine triphosphate (ATP)/adenosine diphosphate (ADP) ratios have been demonstrated in steroidogenic follicular cells of the ovary (111).

## Conclusions

In this study, advances in metabolite identification have led to the profiling of a large number of metabolites in MA-10 Leydig cells; this has enhanced predictive understanding of cellular physiology as presented. There could indeed be metabolomic departures from in vivo Leydig cell physiology distinct from a steroidogenic function in that MA-10 Leydig cells are continuously proliferating with a constant anabolic metabolism. Moreover, protein synthesis for secretions such as Inhibin B (INHBB) and Insulin-like peptide 3 (INSL3) and associated feedback regulations might be disrupted in MA-10 cells. Irrespective of the cell type, limitations to the extent of metabolite annotations are a known bottle neck to the metabolomics approach (112). However, for the different pathways identified and studied, there appears to be conserved expression of the biosynthetic and metabolic genes between MA-10 cells and primary Leydig cells. In addition, impacts on steroidogenic function presents a causal link to interpret metabolite pathway-specific functions in steroidogenesis. Therefore, despite these negatives, our data uncovers some core functional characteristics that advance understanding of the resting homeostasis of steroidogenic Leydig cells.

## Materials and Methods

### Leydig cells

Cultured clonal line of cells from Leydig cell tumors (MA-10 cells) (113) that provide a suitable model system for the study of steroidogenesis was used for experiments. The MA-10 Leydig cell line was maintained at 37°C in an environment of 5% CO_2_ in DMEM high glucose (25 mM glucose, 1 mM pyruvate) containing 10% fetal bovine serum (FBS) and 1% penicillin–streptomycin as previously described (114–116). Two defined LH-responsive MA-10 cell subclones (16), were used for testing steroidogenesis. For both metabolomics and functional testing, cells were plated at ∼50% confluence before use in experiments. Staining for neutral lipids was performed using Nile Red as described previously (117). MA-10 Leydig cells grown on microscope coverslips at >80% confluence were fixed using 4% formaldehyde for 30 minutes at room temperature and then washed with phosphate buffered saline/PBS. The Nile Red stock solution (1 mg/ml in acetone) was diluted to 500 ng/ml with PBS and added to immerse the fixed cells. The cells were then incubated for 15 minutes protected from light at room temperature. The cells were then carefully washed with PBS and mounted using ProLong Gold antifade mounting medium containing 4′,6-diamidino-2-phenylindole/DAPI (Life Technologies). Fluorescence after excitation at 555 nm was visualized under a DMI3000B microscope (Leica) and images were acquired using a monochromatic cooled high-resolution camera, DFC360 FX (Leica).

### Sample processing and preparation

Leydig cell pellets (4e+6 MA-10 cells) were homogenized using a mechanical disrupter (Geno/Grinder). Subsequent sample processing and preparations were performed as previously described (118). In brief, homogenates were extracted by adding 225 µl of cold methanol containing an internal standard mixture [lysophosphatidylethanolamine LPE(17:1), LPC(17:0), PC(12:0/13:0), PE(17:0/17:0), PG(17:0/17:0), d 7-cholesterol, SM(d18:1/17:0), CM Cer(d18:1/17:0), monoacylglycerol MG(17:0/0:0/0:0), DG(12:0/12:0/0:0) and TG d 5-TG(17:0/17:1/17:0), d 3-palmitic acid], and 750 µl of cold methyl tert-butyl ether (MTBE) containing the internal standard cholesteryl ester CE 22:1 and 188 µl of Liquid chromatography-mass spectrometry (LC-MS)grade water. After vortex mixing, samples were centrifuged at 14,000 × g for 2 minutes to separate the extracted phases. The upper hydrophobic fraction (350 µl) was collected for lipid analysis, and the lower aqueous fraction (125 µl) was collected for metabolite analysis. Both fractions were evaporated to dryness using a vacuum centrifuge (Labconco).

### Analysis for lipids

Reverse-phase lipid chromatography–quadrupole/time-of-flight mass spectrometry (CSH–QTOF MS) was performed as previously described (118). The lipid extracted phase was redissolved in a 90:10 methanol:toluene mixture (110 µl) (Fisher Scientific) containing 50 ng/mL CUDA (12-[[(cyclohexylamino)carbonyl]amino]- dodecanoic acid, Cayman Chemical) and analyzed using an Agilent 1290 Infinity LC system (Agilent Technologies). Analysis in both positive and negative ESI modes and different mobile-phase modifiers for each polarity were used to increase the coverage of lipids measured (119). For ESI (+) we used ammonium formate with formic acid as mobile phase modifiers. The addition of formic acid improved detection of CE, DG, and PC lipid classes compared to ammonium formate alone. For ESI (−) we used ammonium acetate as mobile phase modifier. Volumes of 3 and 5 µl used for positive and negative ESI modes, respectively, were injected into an Acquity UPLC CSH C18 column (100  ×  2.1 mm; 1.7 µm) coupled to an Acquity UPLC charged surface hybrid (CSH) C18 VanGuard precolumn (5  ×  2.1 mm; 1.7 µm). The column was maintained at 65°C with a flow rate of 0.6 mL/minute. Mobile phases were prepared with 10 mM ammonium formate and 0.1% formic acid for positive ESI analyses and 10 mM ammonium acetate for negative ESI data acquisition. Both positive and negative ESI modes used identical mobile phase composition of 60:40 acetonitrile:water (Fisher Scientific) for mobile phase A and 90:10 isopropanol:acetonitrile (Fisher Scientific) for mobile phase B. Gradient elution was performed from 0 minute 15% (B), 0 to 2 minute 30% (B), 2 to 2.5 minute 48% (B), 2.5 to 11 minute 82% (B), 11 to 11.5 minute 99% (B), 11.5 to 12 minute 99% (B), 12 to 12.1 minute 15% (B), and 12.1 to 15 minute 15% (B). Lipids were detected and quantified using an Agilent 6550 iFunnel accurate mass quadrupole/time-of-flight (QTOF) mass spectrometer with a jet stream ESI source (Agilent). The QTOF MS instrument was operated in ESI in positive and negative ESI mode with the following parameters: mass range, m/z 50 to 1700; capillary voltage, ±3 kV; nozzle voltage, ±1 kV; gas temperature, 200°C; drying gas (nitrogen), 14 L/min; nebulizer gas (nitrogen), 35 psi; sheath gas temperature, 350°C; sheath gas flow (nitrogen), 11 L/min; acquisition rate, 2 spectra/s. A reference solution (Agilent) was used to correct small mass drifts during the acquisition. Method blanks and human pooled plasma samples were used as QC controls. The quality control check showed that sample injection was not overloading the column. MS-DIAL software (120) was used to process the raw data and lipids were reported only when detected in 50% of samples in each group. Annotations were made based on an accurate mass and retention time lipid library created using LipidBlast, as described previously (121). The percentage of the number of annotated metabolites is calculated relative to the total metabolites identified in each assay.

### Analysis of polar metabolites

Waters Acquity UPLC BEH Amide column (150 mm length x 2.1 mm id; 1.7 μm particle size) with an additional Waters Acquity VanGuard BEH Amide precolumn (5 mm × 2.1 mm id; 1.7 μm particle size) was maintained at 45°C coupled to an Agilent 1290 Infinity UHPLC and used as previously described (122, 123). Five microliters of resuspended sample in starting LG buffer were injected onto the column. The mobile phases were prepared with 10 mM ammonium formate and 0.125% formic acid (Sigma–Aldrich) in either 100% LC-MS grade water for mobile phase (A) or 95:5 v/v acetonitrile:water for mobile phase (B). Gradient elution was performed from 100% (B) at 0 to 2 minute to 70% (B) at 7.7 minutes, 40% (B) at 9.5 minute, 30% (B) at 10.25 minute, 100% (B) at 12.75 minute, isocratic until 16.75 minute with a column flow of 0.4 mL/minute. MS/MS spectra were collected in data-dependent mode using a 5600 + TripleTOF MS (SCIEX, Framingham, MA, USA). Data were collected in ESI(+) mode with the following parameters: m/z 50 to 1700, curtain gas: 35, ion source gas 1 and 2: 60, temperature: 350°C, ion spray voltage floating: +4.5 kV, declustering potential: 80 V, MS1 accumulation time 100 ms, MS2 accumulation time 50 ms, dependent product ion scan number eight, intensity threshold 1000, active precursor exclusion after 2 spectra for 5 s, collision energy 20 eV with 15 eV collision energy spread. Calibration was performed after every ten injections using APCI positive calibration solution to ensure mass accuracy. Metabolites were identified by MS/MS and accurate mass matching against spectral libraries downloaded from MassBank of North America (https://massbank.us).

### Data analysis and representations

The annotated lipids were classified into two major classes, neutral lipids, and plasma membrane lipids. The neutral lipids were further subcategorized into TG, DG, and TC. The percentage expression (peak height) of these subcategories was calculated relative to the total neutral lipid expression. Similarly, plasma membrane lipids were subcategorized into GPLs and SLs, they were further categorized as PC, LPC, GPC, PE, PS, and CMs, and SM respectively. The percentage expression of all these subcategories was calculated relative to the total expression of plasma membrane lipids. All analyses and heatmap representations were performed using *R* (124). Graphs were generated with processed datasets using Prism 8 (GraphPad).

### Functional testing

Using chemical inhibitors that suppress the use of stored pantothenic acid, choline, and betaine, we evaluated their role in MA-10 cell steroidogenesis. For this experiment, LH-responsive MA-10 cell subclones were treated with a PANK inhibitor (PANKi/2,4-dimethyl-N-[3-(methylthio)phenyl]-pyrido[2′,3′:3,4]pyrazolo[1,5-a]pyrimidine-3-propanamide; 10μM and 100μM), choline kinase (CHOK) inhibitor (CHOKi/V-11-0711; 100μM) or BHMT inhibitor (BHMTi/CBHcy; 100μM) for 60 minutes in serum free medium (Dulbecco's modified Eagle medium containing 25 mM glucose and 1 mM pyruvate). Concentrations were selected based on specified ED50 for each of the inhibitors. Steroidogenesis was then induced with human chorionic gonadotropin (hCG; 1.5 IU/ml) for an additional 6 hours in the continued presence of the inhibitors, and cell culture supernatant was collected and stored (in −20˚C) for measuring progesterone levels. Total cellular protein in each well was collected after lysis with sodium dodecyl sulfate (SDS, 1% w/v in water), and measured using bichionic acid (BCA) assay kit (ThermoFisher Scientific).

### Radioimmunoassay

Progesterone levels in cell culture supernatants were measured as previously described (115). In brief, cell culture supernatants were incubated overnight with I^125^-labeled progesterone (MP Bio) and antiprogesterone antibody (125) at 4˚C for competitive binding. A charcoal-dextran suspension was then added and incubated for 10 minutes at 4^˚^C to absorb the free fraction. Samples were then centrifuged at 1700 x g for 10 minutes and the supernatant was collected. Radioactivity in each collected sample was measured using a scintillation counter (Clinigamma Automatic, Wallac). Progesterone levels were estimated relative to detection of an all-encompassing range of progesterone standards–standard curve. The progesterone levels were subsequently normalized to the total protein content of each well. Statistical comparisons were performed comparing controls and each of the treatment concentrations using Student's t test; *P* < 0.05 was considered significant.

### Transcriptomics in primary and MA-10 Leydig cells

Expression of genes associated with the different metabolic pathways was determined using mRNA-sequencing transcriptomics datasets of primary Leydig and MA-10 cells. Transcriptome of adult primary Leydig cells was from Gene Expression Omnibus (GEO) datasets GSE171746 (126) and MA-10 cells were from control samples of Bioproject Accession number PRJNA783636 (127). Using R package EdgeR normalized counts per million (CPM) values were obtained and logarithmic conversions were performed (128). Gene lists associated with the different biochemical pathways were obtained from pathway ontology for mouse annotations within the rat genome database/RGD (URL: http://rgd.mcw.edu/) (129). Heatmaps were generated for comparisons using R function pheatmap.

## Supplementary Material

pgac215_Supplemental_Files

## Data Availability

All data are included in the manuscript and/or Supplementary Material.
