# Supplementary material for: Functional metabolite reserves and lipid homeostasis revealed by the MA-10 Leydig cell metabolome
Source: PNAS Nexus. 2022 Sep 27;1(4):pgac215. doi: 10.1093/pnasnexus/pgac215 (PMC9802464; doi:10.1093/pnasnexus/pgac215)
Supplement: pgac215_Supplemental_Files [file pgac215_supplemental_files.zip › PNASNEXUS-PNASNEXUS-2022-00318-s01.docx]

**Supplementary figures**

**Figure S1.** Gene expression profile of neutral and membrane lipid pathways in primary (Pri) and MA-10 Leydig cells. Genelist heatmaps showing mRNA expression levels for pathways associated with: (A) cholesterol biosynthesis, (B) fatty acid metabolism, (C) sphingolipid metabolism, and (D) glycero-phospholipid metabolism.

**Figure S2.** Transcriptomic profile of amphibolic pathways, coenzyme A, choline-betaine, amino acids and other metabolites in primary (Pri) and MA-10 Leydig cells. Genelist heatmaps showing mRNA expression levels for pathways associated with: (A) glycolysis (B) CoA synthesis (C) TCA cycle (D) Choline-betaine pathway (E) highly expressed amino acids (glutamine, isoleucine, homoserine and aspartate) and creatine pathways.

**Supplementary dataset**

**File 1. MA-10 cell quantitative metabolite profiles. [Sheet I]** Metabolites identified in electrospray ionization (ESI) in positive mode. **[Sheet II]** Metabolites identified in ESI negative mode. **[Sheet III]** Metabolites identified in hydrophilic interaction chromatography (HILIC) mode.
